# Dicyclo­hexyl­bis­(naphthalen-1-ylmeth­yl)phospho­nium chloride chloro­form disolvate

**DOI:** 10.1107/S1600536812046661

**Published:** 2012-11-17

**Authors:** Saravanan Gowrisankar, Helfried Neumann, Anke Spannenberg, Matthias Beller

**Affiliations:** a Leibniz-Institut für Katalyse e. V. an der Universität Rostock, Albert-Einstein-Strasse 29a, 18059 Rostock, Germany

## Abstract

In the title solvated phosphonium salt, C_34_H_40_P^+^·Cl^−^·2CHCl_3_, the two cyclo­hexyl and two 1-naphthyl­methyl groups at the P atom are in a distorted tetra­hedral arrangement [105.26 (6)–113.35 (6)°]. Both cyclo­hexyl rings adopt a chair conformation. The dihedral angle between the naphthyl ring systems is 74.08 (3)°.

## Related literature

A multitude of phospho­nium salts are known in the literature. For some examples of the type [P*R*′_2_ *R*′′_2_]*X* (*R*′ = Me, *R*′′ = Ph, *X* = I), see: Staples *et al.* (1995 ▶); Dornhaus *et al.* (2005 ▶), (*R*′ = Me, *R*′′ = Ph, *X* = Br), see: Staples *et al.* (1995 ▶) and (*R*′ = Me, *R*′′ = fluoren-9-yl, *X* = I), see: Brady *et al.* (2000 ▶).
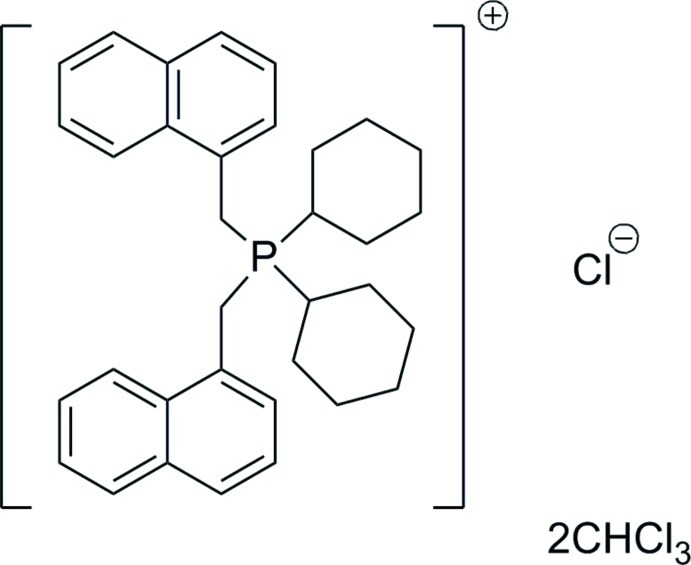


## Experimental

### 

#### Crystal data

C_34_H_40_P^+^·Cl^−^·2CHCl_3_
*M* *_r_* = 753.82
Triclinic, 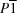

*a* = 12.3079 (2) Å
*b* = 12.7150 (2) Å
*c* = 14.0310 (3) Å
α = 77.785 (1)°
β = 64.242 (1)°
γ = 64.738 (1)°
*V* = 1787.56 (6) Å^3^
*Z* = 2
Mo *K*α radiation
μ = 0.63 mm^−1^
*T* = 150 K
0.43 × 0.30 × 0.19 mm

#### Data collection

Bruker Kappa APEXII DUO diffractometer
Absorption correction: multi-scan (*SADABS*; Bruker, 2008 ▶) *T*_min_ = 0.96, *T*_max_ = 1.00
40097 measured reflections
8871 independent reflections
7562 reflections with *I* > 2σ(*I*)
*R*_int_ = 0.025

#### Refinement

*R*[*F* ^2^ > 2σ(*F* ^2^)] = 0.030
*wR*(*F* ^2^) = 0.080
*S* = 1.03
8871 reflections
397 parameters
H-atom parameters constrained
Δρ_max_ = 0.53 e Å^−3^
Δρ_min_ = −0.44 e Å^−3^

### 

Data collection: *APEX2* (Bruker, 2011 ▶); cell refinement: *SAINT* (Bruker, 2009 ▶); data reduction: *SAINT*; program(s) used to solve structure: *SHELXS97* (Sheldrick, 2008 ▶); program(s) used to refine structure: *SHELXL97* (Sheldrick, 2008 ▶); molecular graphics: *XP* in *SHELXTL* (Sheldrick, 2008 ▶); software used to prepare material for publication: *SHELXTL*.

## Supplementary Material

Crystal structure: contains datablock(s) I, global. DOI: 10.1107/S1600536812046661/pk2454sup1.cif

Structure factors: contains datablock(s) I. DOI: 10.1107/S1600536812046661/pk2454Isup2.hkl

Supplementary material file. DOI: 10.1107/S1600536812046661/pk2454Isup3.cml

Additional supplementary materials:  crystallographic information; 3D view; checkCIF report

## Figures and Tables

**Fig. 1.**
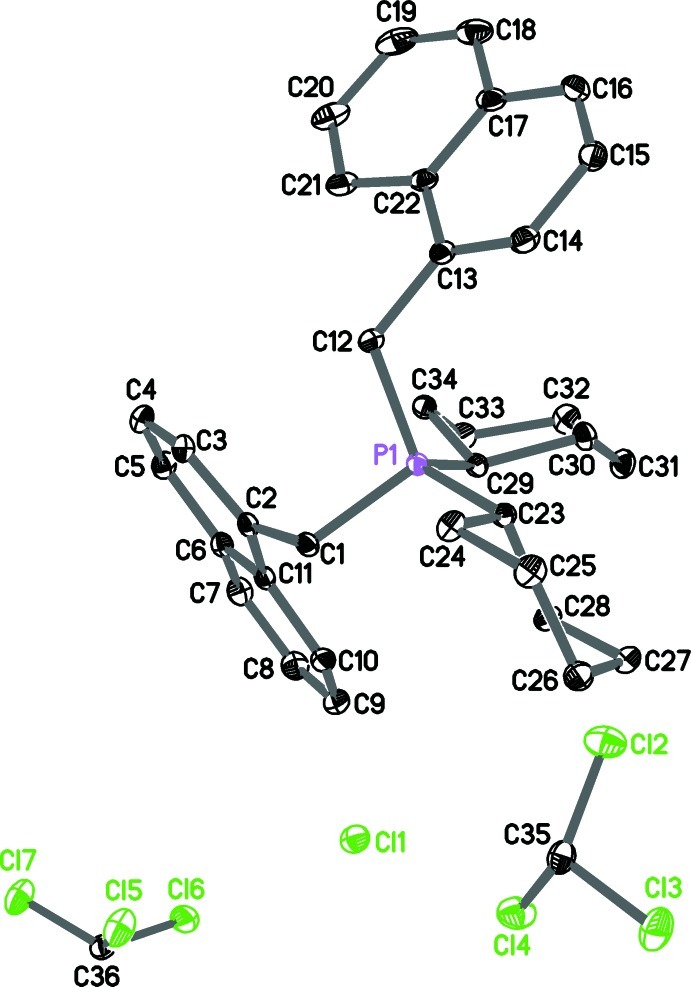
Molecular structure of the title compound with 30% displacement ellipsoids. Hydrogen atoms are omitted for clarity. The asymmetric unit contains besides one molecule of the phosphonium chloride two chloroform solvent molecules.

**Fig. 2.**
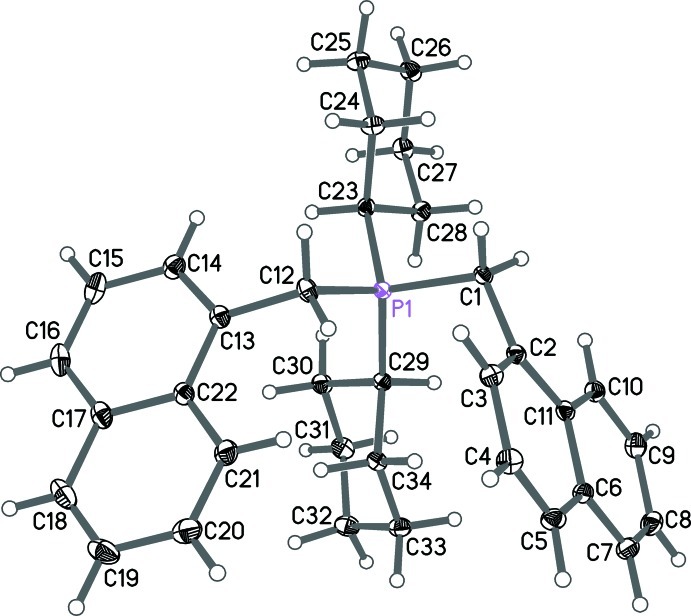
Displacement ellipsoid plot (30%) of the cation [C34H40P]+ with hydrogen atoms depicted as small arbitrary circles.

## supplementary crystallographic information

### Comment

Phosphonium salts are used in a wide range of applications such as ionic liquids, as antimicrobial agents, and as surfactants. Recently, we were interested in the preparation of dicyclohexyl(1-naphthylmethyl)phosphine. During this preparation, dicyclohexylbis(1-naphthylmethyl)phosphonium chloride was formed in a rapid and highly selective manner. We have now observed a reaction of 1-(chloromethyl)naphthalene with Cy_2_PH at 50 °C in acetone which produces the phosphonium salt in nearly quantitative yield. Interestingly, this phosphonium salt is unknown in the literature. Crystals suitable for X-ray crystal structure analysis could be obtained by crystallization from a chloroform/heptane mixture.

The asymmetric unit of the title compound contains besides one unit of the phosphonium chloride, two chloroform molecules (Fig. 1). The phosphorus atom carries two cyclohexyl and two 1-naphthylmethyl groups. Its tetrahedral environment is expressed by C–P–C angles of 105.26 (6) — 113.35 (6)° (Fig. 2). The dihedral angle between the naphthyl groups is 74.08 (3)°. Both cyclohexyl rings adopt the chair conformation.

### Experimental

1-(chloromethyl)naphthalene (4.50 mmol, 795 mg) and dicyclohexylphosphine (2.25 mmol, 500 mg) were dissolved in 4 ml of anhydrous acetone. The solution was stirred at 40 °C overnight. Afterwards acetone was removed *in vacuo*, the residue was dissolved in water and extracted with 50 ml of CHCl_3_. After concentration, 1100 mg (95%) of the title compound were obtained. Colourless crystals suitable for X-ray analysis, were grown from a CHCl_3_/heptane mixture at 8 °C for 4 days. ^1^H NMR (CDCl_3_, internal TMS): δ 8.39–8.36 (m, 1H), 7.83–7.72 (m, 2H), 7.69–7.64 (m, 1H), 7.61–7.53 (m, 1H), 7.49–7.41 (m, 1H), 7.40–7.32 (m, 1H), 4.85 (d, *J* = 15.0 Hz, 2H), 2.77–2.58 (m, 1H), 1.61–1.44 (m, 4H), 1.22–0.75 (m, 6H). ^31^P NMR (CDCl_3_, internal TMS): 32.35. HRMS: calcd. for C_34_H_40_P+: 479.28621; found: 479.28541.

### Refinement

H atoms were placed in idealized positions with d(C—H) = 0.95 Å (C_aromatic_—H), 1.00 Å (C_tertiary_—H), 0.99 Å (CH_2_) and refined using a riding model with *U*_iso_(H) fixed at 1.2 *U*_eq_(C).

### Crystal data

**Table:**

C_34_H_40_P^+^·Cl^−^·2CHCl_3_	*Z* = 2
*M**_r_* = 753.82	*F*(000) = 784
Triclinic, *P*1	*D*_x_ = 1.401 Mg m^−^^3^
*a* = 12.3079 (2) Å	Mo *K*α radiation, λ = 0.71073 Å
*b* = 12.7150 (2) Å	Cell parameters from 9914 reflections
*c* = 14.0310 (3) Å	θ = 2.4–28.8°
α = 77.785 (1)°	µ = 0.63 mm^−^^1^
β = 64.242 (1)°	*T* = 150 K
γ = 64.738 (1)°	Prism, colourless
*V* = 1787.56 (6) Å^3^	0.43 × 0.30 × 0.19 mm

### Data collection

**Table:**

Bruker Kappa APEXII DUO diffractometer	8871 independent reflections
Radiation source: fine-focus sealed tube	7562 reflections with *I* > 2σ(*I*)
Curved graphite monochromator	*R*_int_ = 0.025
Detector resolution: 8.33 pixels mm^-1^	θ_max_ = 28.3°, θ_min_ = 1.6°
ω and φ scans	*h* = −16→16
Absorption correction: multi-scan (*SADABS*; Bruker, 2008)	*k* = −16→16
*T*_min_ = 0.96, *T*_max_ = 1.00	*l* = −18→18
40097 measured reflections	

### Refinement

**Table:**

Refinement on *F*^2^	Primary atom site location: structure-invariant direct methods
Least-squares matrix: full	Secondary atom site location: difference Fourier map
*R*[*F*^2^ > 2σ(*F*^2^)] = 0.030	Hydrogen site location: inferred from neighbouring sites
*wR*(*F*^2^) = 0.080	H-atom parameters constrained
*S* = 1.03	*w* = 1/[σ^2^(*F*_o_^2^) + (0.0389*P*)^2^ + 0.7399*P*] where *P* = (*F*_o_^2^ + 2*F*_c_^2^)/3
8871 reflections	(Δ/σ)_max_ = 0.001
397 parameters	Δρ_max_ = 0.53 e Å^−^^3^
0 restraints	Δρ_min_ = −0.44 e Å^−^^3^

### Special details

**Table:**

Geometry. All e.s.d.'s (except the e.s.d. in the dihedral angle between two l.s. planes) are estimated using the full covariance matrix. The cell e.s.d.'s are taken into account individually in the estimation of e.s.d.'s in distances, angles and torsion angles; correlations between e.s.d.'s in cell parameters are only used when they are defined by crystal symmetry. An approximate (isotropic) treatment of cell e.s.d.'s is used for estimating e.s.d.'s involving l.s. planes.
Refinement. Refinement of *F*^2^ against ALL reflections. The weighted *R*-factor *wR* and goodness of fit *S* are based on *F*^2^, conventional *R*-factors *R* are based on *F*, with *F* set to zero for negative *F*^2^. The threshold expression of *F*^2^ > σ(*F*^2^) is used only for calculating *R*-factors(gt) *etc*. and is not relevant to the choice of reflections for refinement. *R*-factors based on *F*^2^ are statistically about twice as large as those based on *F*, and *R*- factors based on ALL data will be even larger.

### Fractional atomic coordinates and isotropic or equivalent isotropic displacement parameters (Å^2^)

**Table:**

	*x*	*y*	*z*	*U*_iso_*/*U*_eq_	
C1	0.44144 (12)	0.61109 (11)	0.90253 (10)	0.0165 (2)	
H1A	0.5159	0.5375	0.8745	0.020*	
H1B	0.4173	0.6098	0.9798	0.020*	
C2	0.32732 (12)	0.61845 (11)	0.88257 (10)	0.0167 (2)	
C3	0.20342 (12)	0.68760 (12)	0.94598 (10)	0.0197 (2)	
H3	0.1905	0.7182	1.0079	0.024*	
C4	0.09523 (13)	0.71393 (12)	0.92063 (11)	0.0230 (3)	
H4	0.0107	0.7627	0.9648	0.028*	
C5	0.11233 (13)	0.66932 (12)	0.83288 (11)	0.0225 (3)	
H5	0.0402	0.6910	0.8138	0.027*	
C6	0.23635 (12)	0.59097 (11)	0.76938 (10)	0.0186 (2)	
C7	0.25333 (14)	0.54077 (12)	0.68056 (11)	0.0236 (3)	
H7	0.1804	0.5593	0.6634	0.028*	
C8	0.37273 (14)	0.46629 (13)	0.61920 (11)	0.0260 (3)	
H8	0.3829	0.4344	0.5590	0.031*	
C9	0.48129 (14)	0.43644 (12)	0.64492 (11)	0.0250 (3)	
H9	0.5639	0.3830	0.6028	0.030*	
C10	0.46889 (13)	0.48361 (11)	0.72995 (10)	0.0204 (2)	
H10	0.5431	0.4629	0.7460	0.024*	
C11	0.34605 (12)	0.56336 (11)	0.79468 (10)	0.0170 (2)	
C12	0.36962 (12)	0.85580 (11)	0.92346 (10)	0.0202 (2)	
H12A	0.2826	0.8574	0.9382	0.024*	
H12B	0.3797	0.8409	0.9919	0.024*	
C13	0.37027 (12)	0.97536 (11)	0.88234 (10)	0.0189 (2)	
C14	0.46891 (13)	1.00426 (12)	0.87525 (11)	0.0237 (3)	
H14	0.5370	0.9478	0.8953	0.028*	
C15	0.47188 (15)	1.11567 (13)	0.83897 (12)	0.0299 (3)	
H15	0.5414	1.1335	0.8347	0.036*	
C16	0.37468 (15)	1.19780 (13)	0.80997 (12)	0.0297 (3)	
H16	0.3778	1.2722	0.7843	0.036*	
C17	0.26942 (14)	1.17314 (12)	0.81783 (10)	0.0242 (3)	
C18	0.16649 (17)	1.25885 (13)	0.78941 (12)	0.0339 (4)	
H18	0.1702	1.3329	0.7627	0.041*	
C19	0.06345 (16)	1.23673 (14)	0.79980 (13)	0.0361 (4)	
H19	−0.0040	1.2950	0.7800	0.043*	
C20	0.05607 (15)	1.12812 (14)	0.83962 (13)	0.0320 (3)	
H20	−0.0173	1.1137	0.8480	0.038*	
C21	0.15393 (14)	1.04247 (12)	0.86660 (11)	0.0253 (3)	
H21	0.1476	0.9692	0.8930	0.030*	
C22	0.26485 (13)	1.06140 (11)	0.85580 (10)	0.0194 (2)	
C23	0.65067 (12)	0.69625 (11)	0.84201 (10)	0.0163 (2)	
H23	0.6741	0.7663	0.8160	0.020*	
C24	0.64789 (13)	0.66496 (12)	0.95505 (10)	0.0214 (3)	
H24A	0.5826	0.7314	1.0013	0.026*	
H24B	0.6218	0.5974	0.9838	0.026*	
C25	0.78219 (13)	0.63550 (13)	0.95501 (11)	0.0239 (3)	
H25A	0.8033	0.7061	0.9346	0.029*	
H25B	0.7800	0.6102	1.0274	0.029*	
C26	0.88789 (13)	0.53957 (12)	0.87858 (11)	0.0241 (3)	
H26A	0.8724	0.4664	0.9036	0.029*	
H26B	0.9739	0.5262	0.8771	0.029*	
C27	0.88938 (12)	0.57150 (12)	0.76716 (11)	0.0227 (3)	
H27A	0.9569	0.5066	0.7197	0.027*	
H27B	0.9118	0.6411	0.7398	0.027*	
C28	0.75650 (12)	0.59640 (11)	0.76726 (10)	0.0203 (2)	
H28A	0.7359	0.5256	0.7910	0.024*	
H28B	0.7586	0.6182	0.6946	0.024*	
C29	0.50206 (12)	0.75688 (11)	0.70404 (9)	0.0165 (2)	
H29	0.5433	0.6776	0.6747	0.020*	
C30	0.58989 (13)	0.82273 (12)	0.62941 (10)	0.0212 (3)	
H30A	0.5469	0.9060	0.6468	0.025*	
H30B	0.6741	0.7900	0.6379	0.025*	
C31	0.61324 (13)	0.80974 (13)	0.51507 (10)	0.0246 (3)	
H31A	0.6667	0.8541	0.4666	0.030*	
H31B	0.6629	0.7268	0.4968	0.030*	
C32	0.48614 (14)	0.85304 (13)	0.49863 (11)	0.0263 (3)	
H32A	0.5059	0.8339	0.4266	0.032*	
H32B	0.4445	0.9388	0.5038	0.032*	
C33	0.39099 (13)	0.79931 (12)	0.57971 (10)	0.0226 (3)	
H33A	0.4259	0.7153	0.5669	0.027*	
H33B	0.3065	0.8366	0.5714	0.027*	
C34	0.36958 (12)	0.81527 (11)	0.69249 (10)	0.0183 (2)	
H34A	0.3083	0.7794	0.7442	0.022*	
H34B	0.3314	0.8992	0.7068	0.022*	
C35	0.89295 (14)	0.32618 (14)	0.56997 (11)	0.0283 (3)	
H35	0.8467	0.3208	0.6485	0.034*	
C36	0.14817 (15)	−0.01492 (14)	0.26931 (12)	0.0309 (3)	
H36	0.1925	−0.1015	0.2645	0.037*	
Cl1	0.71624 (3)	0.29319 (3)	0.82907 (2)	0.02431 (7)	
Cl2	0.83623 (5)	0.47307 (4)	0.52736 (3)	0.04050 (10)	
Cl3	1.06121 (4)	0.27508 (5)	0.53884 (4)	0.04713 (12)	
Cl4	0.86009 (4)	0.24063 (4)	0.51101 (4)	0.04124 (10)	
Cl5	0.19955 (5)	0.03165 (5)	0.34606 (4)	0.05000 (12)	
Cl6	0.19249 (5)	0.04763 (4)	0.14031 (3)	0.04382 (11)	
Cl7	−0.02108 (4)	0.02160 (5)	0.32916 (4)	0.04959 (12)	
P1	0.49134 (3)	0.73346 (3)	0.84032 (2)	0.01467 (7)	

### Atomic displacement parameters (Å^2^)

**Table:**

	*U*^11^	*U*^22^	*U*^33^	*U*^12^	*U*^13^	*U*^23^
C1	0.0170 (6)	0.0159 (6)	0.0181 (5)	−0.0075 (5)	−0.0085 (5)	0.0031 (4)
C2	0.0170 (6)	0.0171 (6)	0.0173 (5)	−0.0088 (5)	−0.0077 (5)	0.0041 (4)
C3	0.0191 (6)	0.0231 (6)	0.0167 (6)	−0.0103 (5)	−0.0054 (5)	0.0008 (5)
C4	0.0157 (6)	0.0266 (7)	0.0226 (6)	−0.0076 (5)	−0.0039 (5)	−0.0024 (5)
C5	0.0169 (6)	0.0264 (7)	0.0259 (6)	−0.0091 (5)	−0.0099 (5)	0.0011 (5)
C6	0.0187 (6)	0.0200 (6)	0.0195 (6)	−0.0096 (5)	−0.0085 (5)	0.0021 (5)
C7	0.0254 (7)	0.0271 (7)	0.0238 (6)	−0.0131 (6)	−0.0122 (5)	0.0010 (5)
C8	0.0307 (7)	0.0274 (7)	0.0234 (6)	−0.0136 (6)	−0.0098 (6)	−0.0036 (5)
C9	0.0229 (7)	0.0222 (7)	0.0257 (7)	−0.0078 (5)	−0.0050 (5)	−0.0045 (5)
C10	0.0177 (6)	0.0184 (6)	0.0243 (6)	−0.0064 (5)	−0.0082 (5)	−0.0001 (5)
C11	0.0177 (6)	0.0166 (6)	0.0173 (5)	−0.0085 (5)	−0.0067 (5)	0.0029 (4)
C12	0.0170 (6)	0.0158 (6)	0.0205 (6)	−0.0033 (5)	−0.0038 (5)	−0.0011 (5)
C13	0.0186 (6)	0.0171 (6)	0.0163 (5)	−0.0047 (5)	−0.0036 (5)	−0.0032 (4)
C14	0.0200 (6)	0.0232 (7)	0.0257 (7)	−0.0055 (5)	−0.0068 (5)	−0.0069 (5)
C15	0.0263 (7)	0.0296 (8)	0.0324 (7)	−0.0149 (6)	−0.0006 (6)	−0.0127 (6)
C16	0.0350 (8)	0.0198 (7)	0.0262 (7)	−0.0133 (6)	−0.0006 (6)	−0.0037 (5)
C17	0.0292 (7)	0.0175 (6)	0.0164 (6)	−0.0045 (5)	−0.0037 (5)	−0.0034 (5)
C18	0.0422 (9)	0.0198 (7)	0.0242 (7)	−0.0002 (6)	−0.0111 (6)	0.0001 (5)
C19	0.0359 (8)	0.0308 (8)	0.0293 (7)	0.0071 (7)	−0.0182 (7)	−0.0083 (6)
C20	0.0255 (7)	0.0329 (8)	0.0351 (8)	0.0011 (6)	−0.0148 (6)	−0.0163 (6)
C21	0.0228 (7)	0.0210 (6)	0.0302 (7)	−0.0027 (5)	−0.0103 (6)	−0.0093 (5)
C22	0.0210 (6)	0.0166 (6)	0.0160 (5)	−0.0032 (5)	−0.0052 (5)	−0.0050 (4)
C23	0.0154 (5)	0.0160 (6)	0.0180 (6)	−0.0059 (5)	−0.0065 (5)	−0.0013 (4)
C24	0.0183 (6)	0.0269 (7)	0.0181 (6)	−0.0066 (5)	−0.0076 (5)	−0.0023 (5)
C25	0.0221 (6)	0.0284 (7)	0.0248 (6)	−0.0089 (6)	−0.0130 (5)	−0.0007 (5)
C26	0.0187 (6)	0.0226 (7)	0.0304 (7)	−0.0066 (5)	−0.0119 (5)	0.0024 (5)
C27	0.0155 (6)	0.0219 (6)	0.0257 (6)	−0.0049 (5)	−0.0049 (5)	−0.0025 (5)
C28	0.0175 (6)	0.0201 (6)	0.0215 (6)	−0.0051 (5)	−0.0062 (5)	−0.0053 (5)
C29	0.0178 (6)	0.0166 (6)	0.0153 (5)	−0.0070 (5)	−0.0070 (5)	0.0011 (4)
C30	0.0224 (6)	0.0229 (6)	0.0191 (6)	−0.0118 (5)	−0.0079 (5)	0.0043 (5)
C31	0.0232 (7)	0.0280 (7)	0.0180 (6)	−0.0099 (6)	−0.0050 (5)	0.0018 (5)
C32	0.0292 (7)	0.0310 (7)	0.0174 (6)	−0.0109 (6)	−0.0100 (5)	0.0024 (5)
C33	0.0235 (6)	0.0249 (7)	0.0210 (6)	−0.0077 (5)	−0.0116 (5)	−0.0006 (5)
C34	0.0181 (6)	0.0179 (6)	0.0182 (6)	−0.0062 (5)	−0.0078 (5)	0.0013 (5)
C35	0.0259 (7)	0.0374 (8)	0.0219 (6)	−0.0135 (6)	−0.0069 (6)	−0.0039 (6)
C36	0.0313 (8)	0.0293 (8)	0.0281 (7)	−0.0107 (6)	−0.0058 (6)	−0.0078 (6)
Cl1	0.02448 (16)	0.02486 (16)	0.01802 (14)	−0.00554 (13)	−0.00729 (12)	−0.00050 (11)
Cl2	0.0563 (3)	0.0318 (2)	0.0359 (2)	−0.01182 (18)	−0.02168 (19)	−0.00852 (16)
Cl3	0.0290 (2)	0.0641 (3)	0.0494 (3)	−0.0193 (2)	−0.01541 (18)	−0.0004 (2)
Cl4	0.0438 (2)	0.0338 (2)	0.0546 (3)	−0.01270 (18)	−0.0253 (2)	−0.00839 (18)
Cl5	0.0404 (2)	0.0713 (3)	0.0443 (2)	−0.0256 (2)	−0.00782 (19)	−0.0232 (2)
Cl6	0.0471 (2)	0.0364 (2)	0.0358 (2)	−0.01224 (19)	−0.01105 (18)	0.00287 (17)
Cl7	0.0329 (2)	0.0653 (3)	0.0516 (3)	−0.0232 (2)	−0.00251 (19)	−0.0249 (2)
P1	0.01423 (14)	0.01409 (14)	0.01540 (14)	−0.00529 (11)	−0.00604 (11)	0.00047 (11)

### Geometric parameters (Å, º)

**Table:**

C1—C2	1.5095 (16)	C23—C24	1.5403 (17)
C1—P1	1.8262 (12)	C23—C28	1.5405 (17)
C1—H1A	0.9900	C23—P1	1.8204 (12)
C1—H1B	0.9900	C23—H23	1.0000
C2—C3	1.3775 (18)	C24—C25	1.5308 (18)
C2—C11	1.4301 (17)	C24—H24A	0.9900
C3—C4	1.4116 (18)	C24—H24B	0.9900
C3—H3	0.9500	C25—C26	1.522 (2)
C4—C5	1.3603 (19)	C25—H25A	0.9900
C4—H4	0.9500	C25—H25B	0.9900
C5—C6	1.4185 (19)	C26—C27	1.5237 (19)
C5—H5	0.9500	C26—H26A	0.9900
C6—C7	1.4144 (18)	C26—H26B	0.9900
C6—C11	1.4253 (17)	C27—C28	1.5269 (18)
C7—C8	1.362 (2)	C27—H27A	0.9900
C7—H7	0.9500	C27—H27B	0.9900
C8—C9	1.410 (2)	C28—H28A	0.9900
C8—H8	0.9500	C28—H28B	0.9900
C9—C10	1.3685 (19)	C29—C34	1.5433 (17)
C9—H9	0.9500	C29—C30	1.5439 (17)
C10—C11	1.4236 (18)	C29—P1	1.8264 (12)
C10—H10	0.9500	C29—H29	1.0000
C12—C13	1.5097 (17)	C30—C31	1.5344 (18)
C12—P1	1.8124 (13)	C30—H30A	0.9900
C12—H12A	0.9900	C30—H30B	0.9900
C12—H12B	0.9900	C31—C32	1.526 (2)
C13—C14	1.3708 (19)	C31—H31A	0.9900
C13—C22	1.4335 (18)	C31—H31B	0.9900
C14—C15	1.411 (2)	C32—C33	1.5260 (19)
C14—H14	0.9500	C32—H32A	0.9900
C15—C16	1.365 (2)	C32—H32B	0.9900
C15—H15	0.9500	C33—C34	1.5305 (17)
C16—C17	1.413 (2)	C33—H33A	0.9900
C16—H16	0.9500	C33—H33B	0.9900
C17—C22	1.4239 (19)	C34—H34A	0.9900
C17—C18	1.424 (2)	C34—H34B	0.9900
C18—C19	1.355 (3)	C35—Cl4	1.7546 (15)
C18—H18	0.9500	C35—Cl3	1.7571 (15)
C19—C20	1.401 (2)	C35—Cl2	1.7636 (16)
C19—H19	0.9500	C35—H35	1.0000
C20—C21	1.372 (2)	C36—Cl7	1.7548 (16)
C20—H20	0.9500	C36—Cl5	1.7582 (16)
C21—C22	1.4247 (19)	C36—Cl6	1.7632 (16)
C21—H21	0.9500	C36—H36	1.0000
			
C2—C1—P1	111.98 (8)	C25—C24—H24B	109.5
C2—C1—H1A	109.2	C23—C24—H24B	109.5
P1—C1—H1A	109.2	H24A—C24—H24B	108.1
C2—C1—H1B	109.2	C26—C25—C24	111.50 (11)
P1—C1—H1B	109.2	C26—C25—H25A	109.3
H1A—C1—H1B	107.9	C24—C25—H25A	109.3
C3—C2—C11	119.73 (11)	C26—C25—H25B	109.3
C3—C2—C1	118.60 (11)	C24—C25—H25B	109.3
C11—C2—C1	121.46 (11)	H25A—C25—H25B	108.0
C2—C3—C4	121.25 (12)	C25—C26—C27	111.24 (11)
C2—C3—H3	119.4	C25—C26—H26A	109.4
C4—C3—H3	119.4	C27—C26—H26A	109.4
C5—C4—C3	119.85 (12)	C25—C26—H26B	109.4
C5—C4—H4	120.1	C27—C26—H26B	109.4
C3—C4—H4	120.1	H26A—C26—H26B	108.0
C4—C5—C6	120.96 (12)	C26—C27—C28	110.69 (11)
C4—C5—H5	119.5	C26—C27—H27A	109.5
C6—C5—H5	119.5	C28—C27—H27A	109.5
C7—C6—C5	121.09 (12)	C26—C27—H27B	109.5
C7—C6—C11	119.40 (12)	C28—C27—H27B	109.5
C5—C6—C11	119.51 (12)	H27A—C27—H27B	108.1
C8—C7—C6	120.98 (13)	C27—C28—C23	109.78 (10)
C8—C7—H7	119.5	C27—C28—H28A	109.7
C6—C7—H7	119.5	C23—C28—H28A	109.7
C7—C8—C9	120.05 (13)	C27—C28—H28B	109.7
C7—C8—H8	120.0	C23—C28—H28B	109.7
C9—C8—H8	120.0	H28A—C28—H28B	108.2
C10—C9—C8	120.68 (13)	C34—C29—C30	108.06 (10)
C10—C9—H9	119.7	C34—C29—P1	114.87 (8)
C8—C9—H9	119.7	C30—C29—P1	115.54 (9)
C9—C10—C11	120.77 (12)	C34—C29—H29	105.8
C9—C10—H10	119.6	C30—C29—H29	105.8
C11—C10—H10	119.6	P1—C29—H29	105.8
C10—C11—C6	118.10 (11)	C31—C30—C29	108.37 (11)
C10—C11—C2	123.55 (11)	C31—C30—H30A	110.0
C6—C11—C2	118.35 (11)	C29—C30—H30A	110.0
C13—C12—P1	117.85 (9)	C31—C30—H30B	110.0
C13—C12—H12A	107.8	C29—C30—H30B	110.0
P1—C12—H12A	107.8	H30A—C30—H30B	108.4
C13—C12—H12B	107.8	C32—C31—C30	112.35 (11)
P1—C12—H12B	107.8	C32—C31—H31A	109.1
H12A—C12—H12B	107.2	C30—C31—H31A	109.1
C14—C13—C22	119.42 (12)	C32—C31—H31B	109.1
C14—C13—C12	119.94 (12)	C30—C31—H31B	109.1
C22—C13—C12	120.56 (12)	H31A—C31—H31B	107.9
C13—C14—C15	121.69 (13)	C31—C32—C33	112.32 (11)
C13—C14—H14	119.2	C31—C32—H32A	109.1
C15—C14—H14	119.2	C33—C32—H32A	109.1
C16—C15—C14	119.87 (14)	C31—C32—H32B	109.1
C16—C15—H15	120.1	C33—C32—H32B	109.1
C14—C15—H15	120.1	H32A—C32—H32B	107.9
C15—C16—C17	120.65 (13)	C32—C33—C34	110.81 (11)
C15—C16—H16	119.7	C32—C33—H33A	109.5
C17—C16—H16	119.7	C34—C33—H33A	109.5
C16—C17—C22	119.77 (13)	C32—C33—H33B	109.5
C16—C17—C18	121.05 (14)	C34—C33—H33B	109.5
C22—C17—C18	119.17 (14)	H33A—C33—H33B	108.1
C19—C18—C17	121.25 (15)	C33—C34—C29	108.82 (10)
C19—C18—H18	119.4	C33—C34—H34A	109.9
C17—C18—H18	119.4	C29—C34—H34A	109.9
C18—C19—C20	120.16 (14)	C33—C34—H34B	109.9
C18—C19—H19	119.9	C29—C34—H34B	109.9
C20—C19—H19	119.9	H34A—C34—H34B	108.3
C21—C20—C19	120.53 (15)	Cl4—C35—Cl3	110.35 (8)
C21—C20—H20	119.7	Cl4—C35—Cl2	110.55 (8)
C19—C20—H20	119.7	Cl3—C35—Cl2	109.80 (8)
C20—C21—C22	121.23 (14)	Cl4—C35—H35	108.7
C20—C21—H21	119.4	Cl3—C35—H35	108.7
C22—C21—H21	119.4	Cl2—C35—H35	108.7
C17—C22—C21	117.63 (12)	Cl7—C36—Cl5	110.33 (8)
C17—C22—C13	118.54 (12)	Cl7—C36—Cl6	110.53 (9)
C21—C22—C13	123.83 (12)	Cl5—C36—Cl6	110.16 (9)
C24—C23—C28	110.48 (10)	Cl7—C36—H36	108.6
C24—C23—P1	111.40 (8)	Cl5—C36—H36	108.6
C28—C23—P1	111.39 (8)	Cl6—C36—H36	108.6
C24—C23—H23	107.8	C12—P1—C23	110.81 (6)
C28—C23—H23	107.8	C12—P1—C1	105.26 (6)
P1—C23—H23	107.8	C23—P1—C1	107.20 (6)
C25—C24—C23	110.61 (10)	C12—P1—C29	113.35 (6)
C25—C24—H24A	109.5	C23—P1—C29	110.05 (6)
C23—C24—H24A	109.5	C1—P1—C29	109.88 (6)
			
P1—C1—C2—C3	−82.43 (12)	C20—C21—C22—C17	1.1 (2)
P1—C1—C2—C11	92.37 (12)	C20—C21—C22—C13	−179.82 (13)
C11—C2—C3—C4	−5.77 (19)	C14—C13—C22—C17	3.00 (18)
C1—C2—C3—C4	169.12 (12)	C12—C13—C22—C17	179.68 (11)
C2—C3—C4—C5	0.9 (2)	C14—C13—C22—C21	−176.03 (13)
C3—C4—C5—C6	3.7 (2)	C12—C13—C22—C21	0.64 (19)
C4—C5—C6—C7	177.41 (13)	C28—C23—C24—C25	56.37 (14)
C4—C5—C6—C11	−3.3 (2)	P1—C23—C24—C25	−179.26 (9)
C5—C6—C7—C8	179.46 (13)	C23—C24—C25—C26	−54.96 (15)
C11—C6—C7—C8	0.2 (2)	C24—C25—C26—C27	55.49 (15)
C6—C7—C8—C9	1.2 (2)	C25—C26—C27—C28	−57.30 (15)
C7—C8—C9—C10	−1.5 (2)	C26—C27—C28—C23	58.46 (14)
C8—C9—C10—C11	0.3 (2)	C24—C23—C28—C27	−58.14 (14)
C9—C10—C11—C6	1.03 (19)	P1—C23—C28—C27	177.48 (9)
C9—C10—C11—C2	−177.99 (12)	C34—C29—C30—C31	−62.81 (13)
C7—C6—C11—C10	−1.30 (18)	P1—C29—C30—C31	166.94 (9)
C5—C6—C11—C10	179.43 (12)	C29—C30—C31—C32	56.70 (15)
C7—C6—C11—C2	177.78 (12)	C30—C31—C32—C33	−51.85 (16)
C5—C6—C11—C2	−1.49 (18)	C31—C32—C33—C34	52.53 (16)
C3—C2—C11—C10	−175.03 (12)	C32—C33—C34—C29	−59.07 (14)
C1—C2—C11—C10	10.23 (18)	C30—C29—C34—C33	64.66 (13)
C3—C2—C11—C6	5.94 (17)	P1—C29—C34—C33	−164.72 (9)
C1—C2—C11—C6	−168.79 (11)	C13—C12—P1—C23	75.13 (11)
P1—C12—C13—C14	−70.57 (15)	C13—C12—P1—C1	−169.30 (10)
P1—C12—C13—C22	112.77 (12)	C13—C12—P1—C29	−49.18 (12)
C22—C13—C14—C15	−2.2 (2)	C24—C23—P1—C12	59.03 (10)
C12—C13—C14—C15	−178.90 (12)	C28—C23—P1—C12	−177.12 (9)
C13—C14—C15—C16	0.1 (2)	C24—C23—P1—C1	−55.33 (10)
C14—C15—C16—C17	1.2 (2)	C28—C23—P1—C1	68.52 (10)
C15—C16—C17—C22	−0.3 (2)	C24—C23—P1—C29	−174.80 (9)
C15—C16—C17—C18	178.96 (14)	C28—C23—P1—C29	−50.95 (10)
C16—C17—C18—C19	−178.01 (14)	C2—C1—P1—C12	75.77 (10)
C22—C17—C18—C19	1.3 (2)	C2—C1—P1—C23	−166.20 (9)
C17—C18—C19—C20	0.3 (2)	C2—C1—P1—C29	−46.61 (10)
C18—C19—C20—C21	−1.2 (2)	C34—C29—P1—C12	−42.18 (11)
C19—C20—C21—C22	0.5 (2)	C30—C29—P1—C12	84.71 (10)
C16—C17—C22—C21	177.34 (12)	C34—C29—P1—C23	−166.90 (9)
C18—C17—C22—C21	−1.99 (18)	C30—C29—P1—C23	−40.01 (11)
C16—C17—C22—C13	−1.76 (18)	C34—C29—P1—C1	75.27 (10)
C18—C17—C22—C13	178.92 (12)	C30—C29—P1—C1	−157.84 (9)
